# Effects of adaptation measures to extreme heat throughout medium and high temperature periods, case study at the Osaka expo site

**DOI:** 10.1038/s41598-024-56458-8

**Published:** 2024-03-11

**Authors:** Hideki Takebayashi, Nao Maeda

**Affiliations:** Department of Architecture, Graduate School of Engineering, Kobe University, Rokkodai 1-1, Nada, Kobe, Hyogo 657-8501 Japan

**Keywords:** Environmental impact, Environmental impact

## Abstract

For the Osaka-Kansai Expo, to be held from April to October 2025, it is necessary to manage the exhibition site with consideration for countermeasures against heat in order to make safe and active use of the outdoor space. In this study, we compared meteorological data observed at Expo site on the sea coast, and at the Osaka Meteorological Observatory in the city center to analyze the quantitative relationship between meteorological elements at both locations, calculated thermal environment indices using these meteorological data, and considered the effects of implementing adaptation measures against the heat. The effect of solar radiation shading is predominant in lowering SET* and WBGT. During the medium temperature period, before mid-June and after mid-September, solar radiation shading avoids uncomfortable conditions, and during the high temperature period, from late June to mid-July and mid-August to early September, solar radiation shading, surface cover improvement, and mist spraying reduce the risk of heat stroke. However, during the extremely hot period, from late July to early August, the risk of heat stroke cannot be avoided by any of the adaptation measures to heat.

For the Osaka-Kansai Expo, to be held from April to October 2025, it is necessary to manage the exhibition site with consideration for countermeasures against heat in order to make safe and active use of the outdoor space. Consideration of heat and cold is essential when organizing large-scale events that utilize outdoor space, such as the Olympics and other sporting events and expositions. For example, the marathon race of the 2021 Tokyo Olympics was held in Sapporo, Hokkaido, approximately 800 km north of Tokyo, in consideration of the heat. Also, since the marathon race was originally planned to be held in Tokyo, highly reflective pavement was installed on the marathon course. Vanos et al.^1^ predicted the thermal comfort of runners and spectators on the marathon course of the Tokyo Olympics, and discussed the possibility of improving the thermal environment by solar radiation shading and other measures. Honjo et al.^2^ conducted a thermal environment evaluation of the 2020 Olympic marathon course using WBGT and UTCI as indices. Bureau of Environment, Tokyo Metropolitan Government^3^ launched in 2017 the “Project to promote heat countermeasures for Tokyo 2020” to mitigate the heat felt by people in high-profile areas where many tourists and visitors gather around the Tokyo 2020 Games venues. 20 cool areas have been created in total by fiscal year 2019. Mist spraying accounted for the largest number of applications (17 of 20), followed by solar radiation shading measures such as parasols, trees, pergolas, awnings, tents, and pavilions (12 of 20). Many of them are used in combination. Other measures included highly reflective pavement, cool benches, plantings, cool air blowers, water-retaining pavement, and reflective film.

Appropriate measures against heat require the proper selection of exhibition site and the effective implementation of adaptation measures to heat. In Tokyo, Osaka, and Nagoya, Takebayashi^4^ found that the thermal environment indices in the outdoor space were mitigated during the summer daytime, especially in coastal areas, and that the air conditioning load was also reduced. The Expo site is located near the coast and is likely to benefit from the sea breezes that prevail during sunny summer days. Takebayashi et al.^5^ conducted a social experimental study on the implementation of various adaptation measures to the heat in the central area of Kobe City, and pointed out the effectiveness of the introduction of solar radiation shading and mist spraying. The Expo Association is actively coordinating the implementation of adaptation measures to the heat at the Expo site. In this study, we compared meteorological data observed at Expo site (Yumeshima Island) on the sea coast, and at the Osaka Meteorological Observatory in the city center to analyze the quantitative relationship between meteorological elements at both locations, calculated thermal environment indices using these meteorological data, and considered the effects of implementing adaptation measures against the heat. Although meteorological conditions at the Expo site can be predicted from the results of previous studies using detailed meteorological observations and numerical simulations, on-site meteorological observations are the most reliable method of prediction. However, conducting measurements at the Expo site is subject to various restrictions. In this context, this data is recognized as valuable observation data, although for a limited period of time. The observation results obtained at the Expo site where no buildings are clearly inconsistent with those obtained at the Expo site where pavilions and other structures have been constructed. However, it is possible to recognize air temperature and humidity characteristics as spatial differences by comparing them with existing meteorological station. Wind velocity and direction, and solar radiation characteristics can be recognized at heights unaffected by ground level buildings. From this standpoint, we analyzed air temperature, humidity, upper-air wind velocity and direction, and solar radiation. At the ground level, wind velocity is assumed to be a fixed value, and thermal environmental indices are calculated assuming ground surface cover and heat countermeasure technology.

## Comparison of meteorological elements at city center and coastal expo site

**Figure 1:**
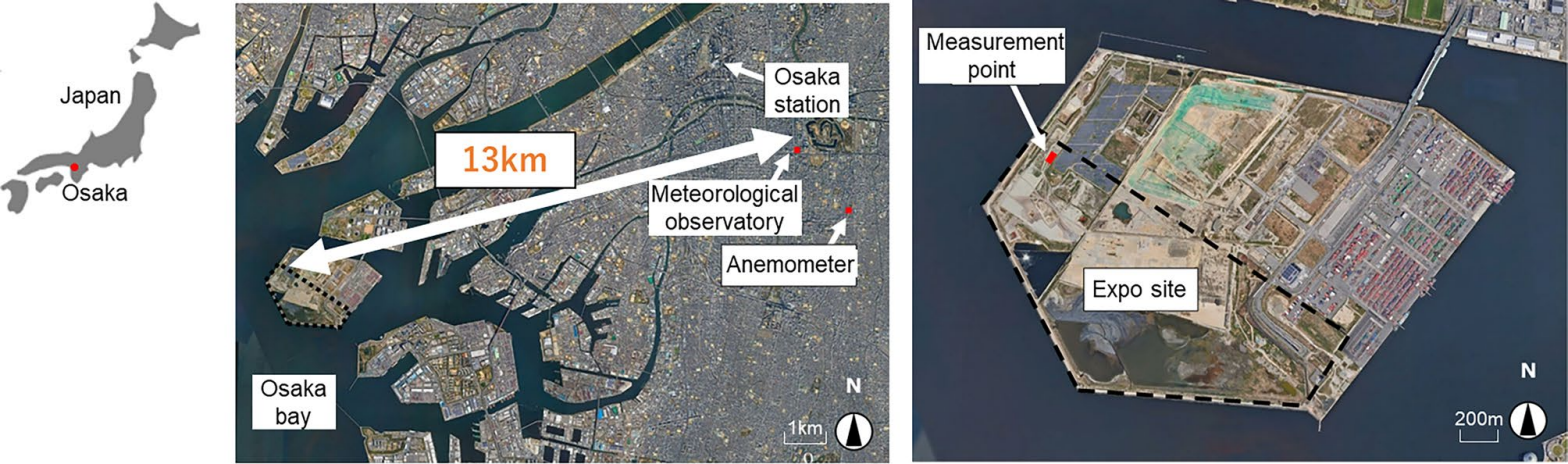
Observation points at Osaka Meteorological Observatory and Expo site.

**Figure 2:**
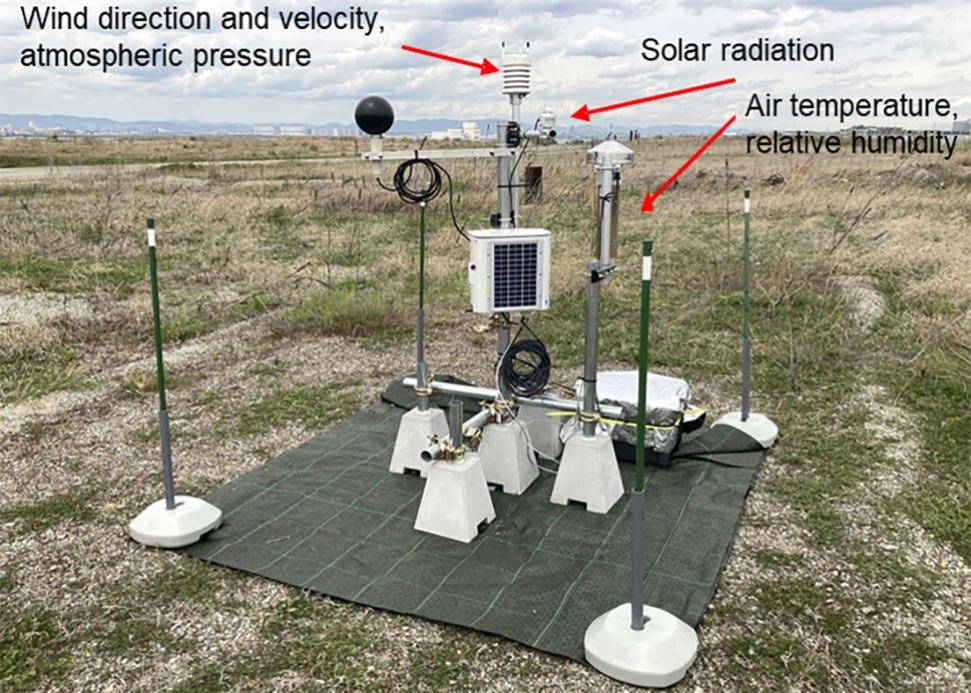
The measurement conditions at Expo site.

The observation points at the Osaka Meteorological Observatory and Expo site are shown in Fig. 1. The distance between the two points is approximately 13 km. The data from the Osaka Meteorological Observatory are hourly values for atmospheric pressure, solar radiation, air temperature, wind velocity, wind direction, relative humidity, cloud cover, sunshine duration, and precipitation. The anemometer was installed on the roof of a building in the city center at a height of 24 m above the ground. The other instruments are located in the open-air field above the ground. The height of the thermo-hygrometer is 1.5 m above the ground. The instruments on Expo site were installed at a height of about 1.5 m above the ground in a open field (bare ground with sparse vegetation). Wind velocity, wind direction, atmospheric pressure, solar radiation, air temperature, and relative humidity were measured. Figure 2 shows the measurement conditions. The thermo-hygrometer was installed in a forced ventilation tube that shielded the solar radiation.

**Figure 3:**
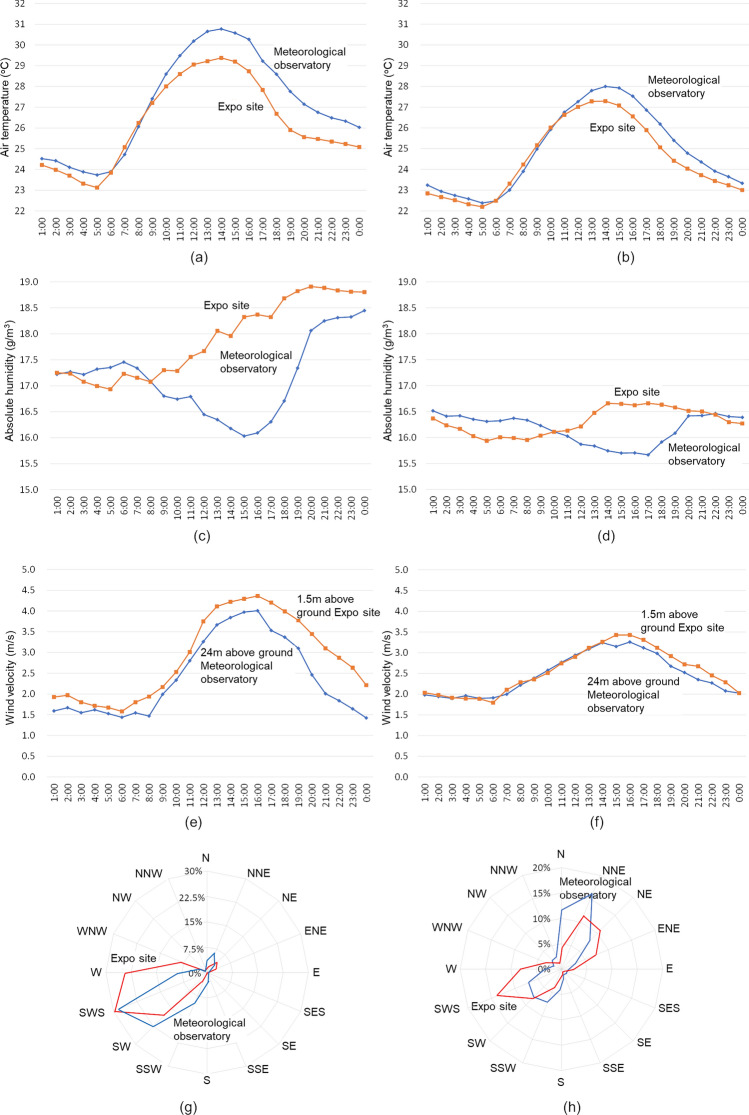
Diurnal air temperature, absolute humidity, wind velocity changes and wind roses averaged on sea-breeze and sunny days and on the other days.

Observation data from April 20 to October 5, 2022 at both points were compared by classifying them into sea-breeze and sunny days and other days. On sea-breeze and sunny days as shown in left sides of Fig. 3, air temperature was 1.4 °C lower, absolute humidity was 1.9 g/m^3^ higher, wind velocity was 0.7 m/s higher, and westerly component is slightly larger at Expo site than at the meteorological observatory. On the other days as shown in right sides of Fig. 3, air temperature was 0.8 °C lower, absolute humidity was 0.8 g/m^3^ higher, wind velocity was 0.2 m/s higher, and west to southwest component is slightly larger at Expo site than at the meteorological observatory. The difference between the both points is clearer on sea-breeze and sunny days than on other days, although it cannot be directly compared between the two periods because of the different weather conditions on those two periods. According to the Osaka Meteorological Observatory's report “Weather in the Kinki Region in 2022”,^6^ the annual mean temperature in 2022 was higher than normal (+ 0.6 °C), with less precipitation in May and June. Therefore, these data represent the situation under more severe weather conditions. Oku and Masumoto^7^ analyzed the spatiotemporal characteristics of air temperature measured by thermometers installed in 60 elementary schools in Osaka City. They pointed out that in a typical summer daytime air temperature distribution, positive anomalies are large in the inland eastern part, and negative anomalies are large in the coastal western part, and air temperature difference between inland and coastal areas reached 2.2 °C. They confirmed that sea breezes with lower air temperatures than on land prevailed during the summer daytime, and that the cooling effect due to cooler air temperature advection was greatest in coastal areas. They pointed out that the effect became smaller the further inland, due to heating from the ground surface and mixing with the relatively hot air over land. Note that the wind velocity at the meteorological observatory is 24 m above the ground on the roof of the building, while that at Expo site is 1.5 m above the ground. Wind direction at Expo site was sea breezy, but was not sea breezy at the meteorological observatory at some times. However, wind directions at both points matched about 60% (within a range of ± 22.5 degrees), regardless of sea-breeze and sunny days. The solar radiation at the two points were almost the same.

## Calculation results of thermal environment indices throughout medium and high temperature periods

The guideline by the Ministry of the Environment of Japan^8^ states that standard effective temperature (SET*) is an index related to the human comfort sensation and Wet Bulb Globe Temperature (WBGT) is an index related to the risk of heat stroke. Since the Heat countermeasure guideline in the city by the Ministry of the Environment of Japan recommends the use of WBGT and SET* as thermal environment indices in outdoor spaces^8^, we used these indices so that many Japanese engineers involved in the operation of the Expo site can make use of the results of this study. While SET*, which is used to assess comfort, is unfortunately a domestic indicator, WBGT, which is used to assess risk, is considered to correspond to a global standard. Since the evaluation period includes slightly cooler periods, the amount of clothing may also change in the morning and evening. Since foreigners, the elderly, and children are included in the evaluation subjects, the metabolic rate and the morphological coefficients with the surroundings change along with the amount of clothing. In order to focus on the inter-comparison of countermeasure technologies in this study, typical values for these parameters are set for Japanese adults in summer^8^. The amount of clothing and metabolic rate were set to 0.6 clo and 2.0 Met, respectively, assuming a warm season and walking conditions. Mean Radiant Temperature (MRT) was calculated assuming that the ground surface cover was normal concrete and the human body was a sphere, and that shading by surrounding objects was not assumed and solar radiation was considered. SET* was calculated using a two-node model of the human body, and WBGT was calculated from the following Eq. (1).

1 $${\text{WBGT }} = \, 0.{\text{7 Tw }} + \, 0.{\text{2 Tg }} + \, 0.{\text{1 Td}}$$

where Tw is wet bulb temperature (°C), Tg is globe temperature (°C), and Td is dry bulb temperature (°C). Tw is calculated from air temperature and relative humidity. Tg is calculated from MRT, air temperature and wind velocity.

According to the study results by Ishii et al.^9^, conditions with SET* exceeding 30.8 °C above the criterion for "slightly uncomfortable" were defined as thermally uncomfortable. According to the heat stroke prevention guidelines in Japan (Japanese Society of Biometeorology^10^, Japan Sport Association^11^), conditions with WBGT exceeding 28 °C above the criterion for "severe alert" were defined as dangerous in terms of heat stroke risk. As a result, SET* ≥ 30.8 °C and WBGT ≥ 28 °C were classified as dangerous, SET* ≥ 30.8 °C and WBGT < 28 °C were not dangerous but uncomfortable, and SET* < 30.8 °C and WBGT < 28 °C were not uncomfortable.

**Figure 4:**
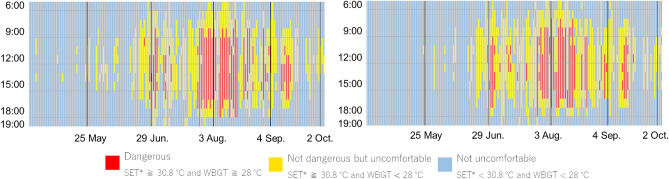
Classification results for dangerous, not dangerous but uncomfortable, and not uncomfortable between 6:00 and 19:00, Left: Osaka Meteorological Observatory, Right: Expo site.

The calculated SET* and WBGT results for both sites were similar, or slightly lower at Expo site than at the meteorological observatory. The classification results for dangerous, not dangerous but uncomfortable, and not uncomfortable between 6:00 and 19:00 at Expo site and the meteorological observatory are shown in Fig. 4. At both sites, dangerous hours are concentrated around maximum temperatures from mid-July to mid-August, while not dangerous but uncomfortable hours are observed during the daytime from mid-June to early October. Although the dangerous hours at Expo site are 44 h less than at the meteorological observatory, it may be necessary to consider restricting the use of outside space during the daytime in August.

## Discussion on adaptation measures to heat

The effects of introducing solar radiation shading, ground surface cover improvement, and mist spraying were examined as typical adaptation measures. Solar radiation shading (e.g., sunshade, parasol, tree shade) has a major effect on direct solar radiation shading, ground surface cover improvement (e.g., greening, water retention, high reflection) has a major effect on ground surface temperature reduction, and mist spraying has a major effect on air temperature reduction and absolute humidity increase. The range of changes in meteorological elements was defined with reference to the Heat Countermeasure Guideline in the City by Ministry of the Environment of Japan^6^.

**Figure 5:**
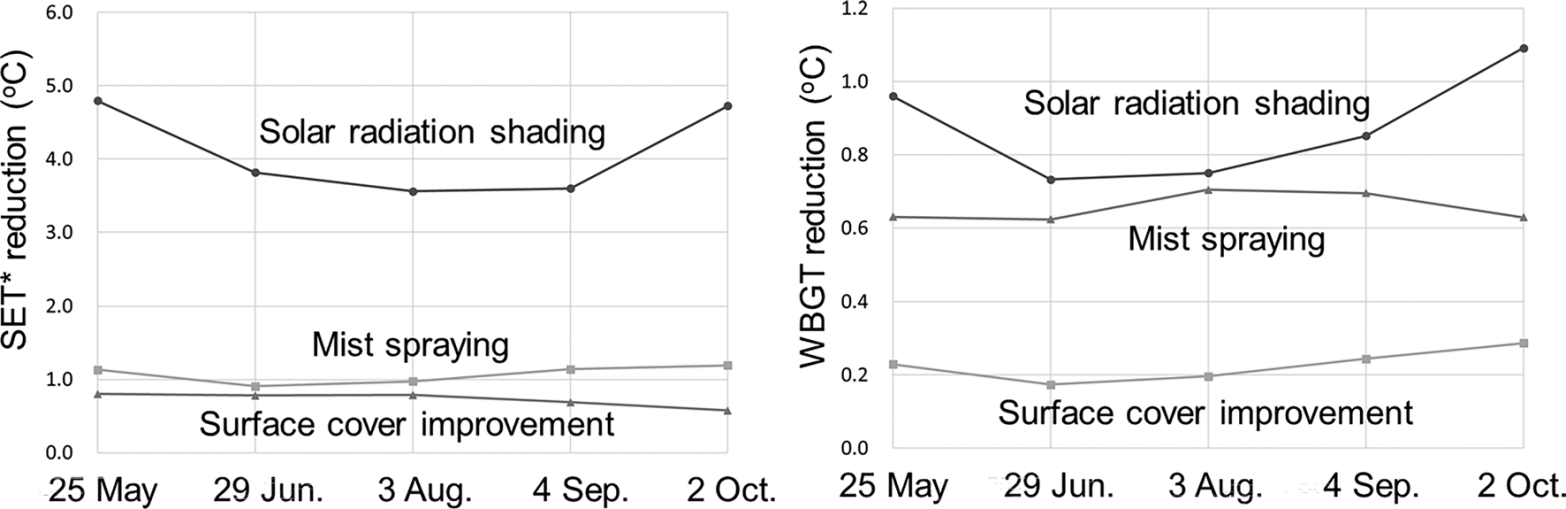
SET* and WBGT reduction at 13:00 on typical sunny days when the adaptation measures were implemented. The typical sunny days which are also shown in Fig. 6, are selected to be representative of the weather conditions on the days before and after.

The thermal environment mitigation effect by each countermeasure technology changes depending on parameters that indicate the characteristics of each technology, such as solar radiation shading rate. Therefore, we conducted the sensitivity analysis shown in Fig. 8 in advance, and as a result, it was confirmed that the difference in effectiveness between each technology was significant. A simulation was conducted with typical parameters set as 100% solar radiation shading rate, 2 °C air temperature reduction with 0.8 g/m^3^ humidity increase, and 15 °C surface temperature reduction, that can be expected to have a relatively large effect. Although the influence process differs depending on each countermeasure technology, such as changing the incident solar radiation to the human body, changing the heat exchange between the human body and the surrounding air, and changing the infrared radiation to the human body, this study provides the evaluation method of each countermeasure technology in the method section and then integrates their effects by using thermal evaluation indices to achieve inter-comparison between them. Figure 5 shows SET* and WBGT reduction at 13:00 on typical sunny days when the adaptation measures were implemented. The difference of the effects by season is not so large. Approximately, the effects of solar radiation shading were 4 °C and 0.9 °C for SET* and WBGT, respectively. Even though surface temperature reduction was 15 °C, SET* and WBGT reductions due to the improved ground surface cover by reducing infrared radiation via the view factor to the human body were as small as 1.1 °C and 0.2 °C, respectively. MRT and Tg decreased by 22.7 °C and 4.3 °C, respectively, due to solar radiation shading, but by 5.8 °C and 1.1 °C, respectively, due to 15 °C decrease in ground surface temperature. The effects of mist spray on air temperature decrease and humidity increase vary greatly depending on the distance between the nozzle and the human body and the ambient wind velocity. Assuming air temperature decrease by 2 °C and humidity increase by 0.8 g/kg’, SET* and WBGT decreased by 0.8 °C and 0.6 °C, respectively. The effect of mist spray was reflected in the decrease in Tg, which was 1.6 °C lower under this condition, as well as in 2 °C decrease in Td. On May 24 and October 2, when wind velocities were low, Tg reduction due to solar radiation shading was as large as 4.8 °C and 5.5 °C, respectively, and WBGT reduction was also large. The effect on SET* is also large in similar cases. The above results show that the effect of solar radiation shading was greater than that of ground cover improvement and mist spraying, which is consistent with the results of previous studies (Takebayashi et al.^5^, Ministry of the Environment of Japan^6^).

**Figure 6:**
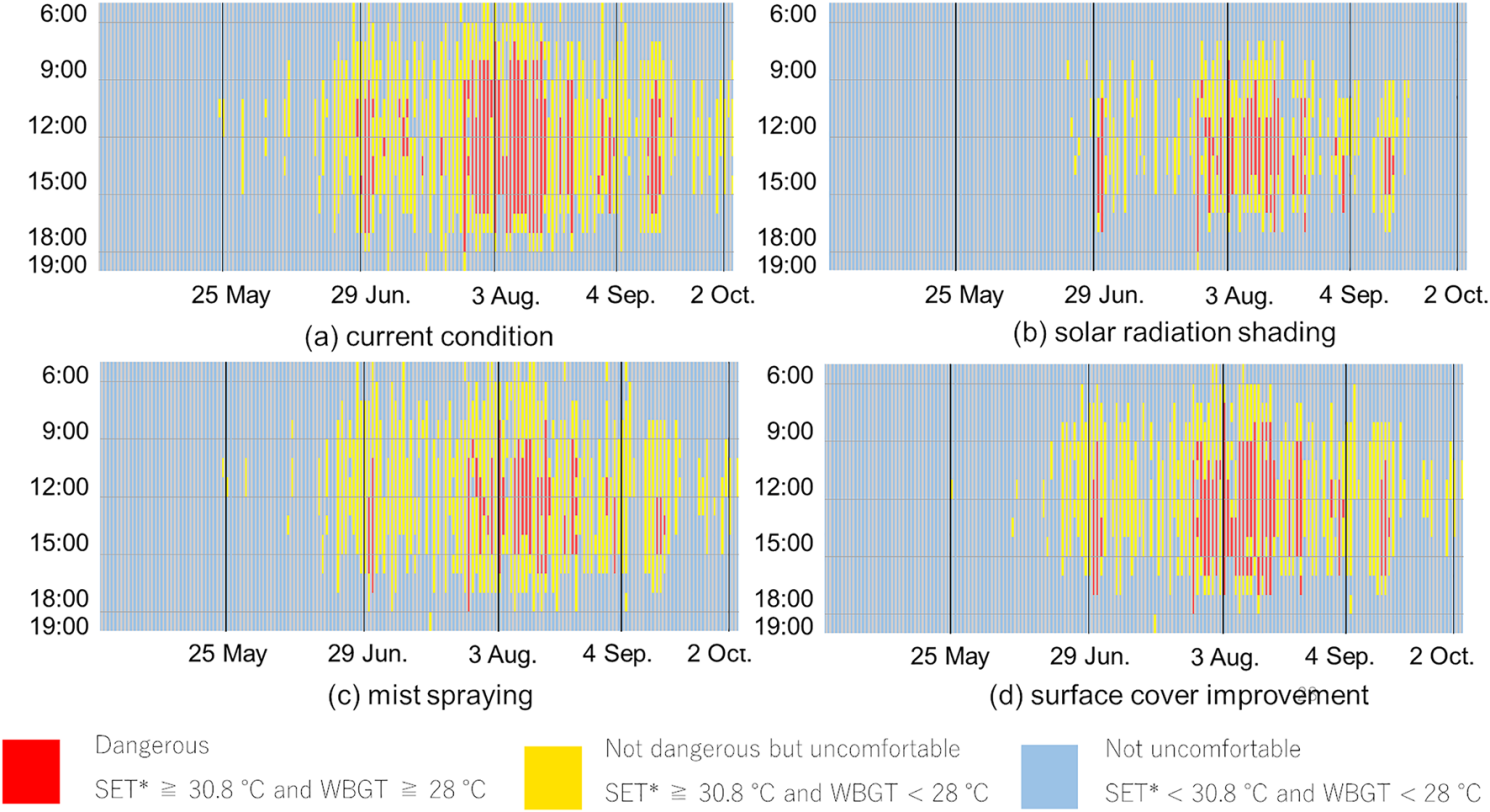
Classification results for dangerous, not dangerous but uncomfortable, and not uncomfortable between 6:00 and 19:00, when adaptation measures to heat are implemented.

This study adapts the evaluation methods for countermeasure technologies to heat considered in previous studies presented in the methods section to the meteorological conditions expected at the Expo site, so it is obvious that the effects of these technologies follow the results of the previous studies. The focus of the discussion is on the effect of countermeasure technologies on the Expo site throughout the duration of the Expo. Classification results for dangerous, not dangerous but uncomfortable, and not uncomfortable between 6:00 and 19:00, when adaptation measures to heat are implemented are shown in Fig. 6. In comparison to the current condition, solar radiation shading reduced dangerous hours by 2 to 6 h per day and non-dangerous but uncomfortable hours by 1 to 4 h per day. Mist spraying also reduced the dangerous time by 1 to 4 h per day, but surface cover improvement did not reduce it so much. On the representative days, solar radiation shading avoided uncomfortable conditions on May 24, June 29, and October 2, and the risk of heat stroke was avoided on June 29 and September 4 due to solar radiation shading, ground surface cover improvement, and mist spraying. However, the discomfort condition could not be avoided by any of the measures on August 3.

The effect of solar radiation shading is predominant in lowering SET* and WBGT. During the medium temperature period, before mid-June and after mid-September, solar radiation shading avoids uncomfortable conditions, and during the high temperature period, from late June to mid-July and mid-August to early September, solar radiation shading, surface cover improvement, and mist spraying reduce the risk of heat stroke. However, during the extremely hot period, from late July to early August, the risk of heat stroke cannot be avoided by any of the adaptation measures to heat. It is necessary to consider the management of the exhibition site with such a thermal environment for visitors assumed. This study has the limitation that the mechanism of countermeasure technologies against heat is reproduced in a simplified form and does not reflect the complex reality of the actual phenomenon.

## Methods

### Calculation method of ground surface temperature

**Figure 7:**
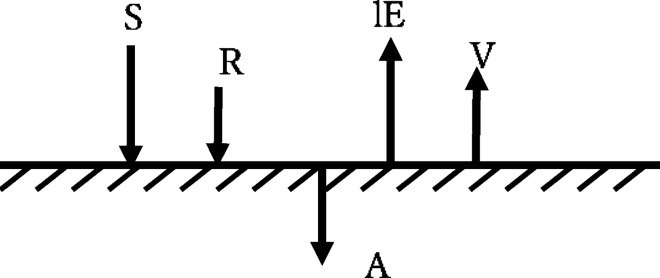
Heat budget components on ground surface.

At the ground surface, each heat budget component shown in Fig. 7 is balanced at a specific ground surface temperature, so the ground surface temperature can be calculated by solving the heat budget equation (Eq. 2)

2 $${\text{S}} + {\text{R}} + {\text{V}} + {\text{A}} + 1{\text{E}} = 0$$

where S is absorbed solar radiation (W/m^2^), R is infrared radiation balance (W/m^2^), V is convective heat flux (W/m^2^), A is conduction heat flux into the ground (W/m^2^), lE is latent heat flux by evapotranspiration (W/m^2^).

## Calculation method of Mean Radiant Temperature (MRT)

The mean radiant temperature is the temperature of the blackbody radiation emitted equivalent to the mean value of the radiant heat received from the surroundings in all directions. The mean radiant temperature MRT when solar radiation is accounted for is as following Eq. (3).

3 $${\text{MRT }} = \, \left( {{\text{aQ}}/\sigma \, + \, \sum {\Phi_{{\text{i}}}\upvarepsilon _{{\text{i}}} {\text{T}}_{{\text{i}}}^{{4}} } } \right)^{{{1}/{4}}}$$

where a is solar radiation absorptance of the human body (-), Q is solar radiation incident on the human body (W/m^2^), σ is Stefan-Boltzmann constant (5.67 × 10^–8^ W/(m^2^K^4^)), Φ is view factor of the sky, walls, and ground surface, ε is the emissivity, assumed to be 1. T is surface temperature of the sky, walls, and ground surface (K). Q is given by the observed values. T of the ground surface is calculated from Eq. (2), and no wall is assumed at this time. Thus, the view factor of the sky and ground surface is 0.5, respectively. T of the sky is calculated by Brunt’s equation using the observed values of air temperature and relative humidity.

## Calculation method of standard effective temperature (SET*)

The standard effective temperature, one of the thermal environment indices, is based on the wetness ratio of the human body and the mean skin temperature, using the six main factors of thermal sensation such as air temperature, MRT, wind velocity, relative humidity, amount of clothing, and metabolic rate. Since the wetness ratio of the human body cannot be easily estimated, SET* is calculated using a two-node model of physiological control model in which the human body is divided into a central part and a skin part to calculate the wetness ratio.

## Calculation method of wet bulb globe temperature (WBGT)

Wet bulb globe temperature is a heat index proposed to prevent heat stroke. The WBGT is based on the heat exchange between the human body and the outside air, and incorporates humidity, solar and infrared radiation, and air temperature, which have a significant influence on the heat balance of the human body. The WBGT for outdoors is calculated by the following Eq. (4).

4 $${\text{WBGT }} = \, 0.{\text{7 Tw }} + \, 0.{\text{2 Tg }} + \, 0.{\text{1 Td}}$$

where Tw is wet bulb temperature (°C), Tg is globe temperature (°C), and Td is dry bulb temperature (°C). Wet bulb temperature Tw is calculated by Sprung's equation from air temperature and relative humidity. Tg is calculated from MRT, Td and wind velocity v (m/s) as following Eq. (5).

5 $${\text{Tg }} = \, \left( {{\text{MRT }} + { 2}.{\text{37 v}}^{{0.{5}}} {\text{Td}}} \right)/\left( {{1 } + { 2}.{\text{37 v}}^{{0.{5}}} } \right)$$

## Estimation method of SET* and WBGT reductions due to adaptation measures to heat

### Solar radiation shading

**Figure 8:**
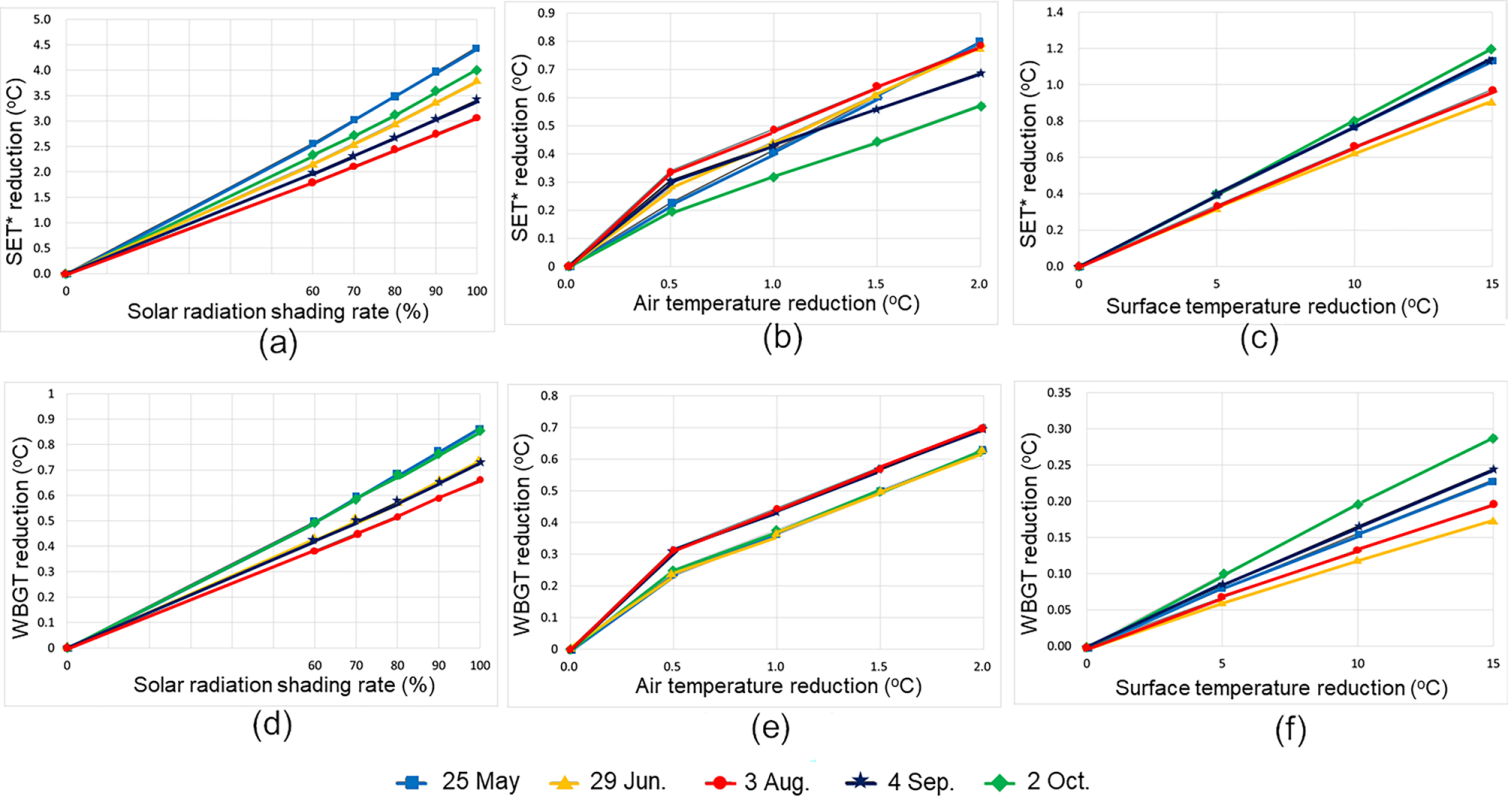
SET* and WBGT reductions due to adaptation measures to heat on representative days assuming the time of maximum air temperature.

MRT reduction is calculated by the decrease in Q (solar radiation incident on the human body) in Eq. (3), according to the increase in solar radiation shading rate. Tg reduction is calculated by Eq. (5). Then, SET* and WBGT reductions are calculated by using MRT and Tg reductions. When solar radiation shading is implemented, the diffuse solar radiation also changes, although the main effect is direct solar radiation shielding. And, the infrared radiation also changes depending on the ground surface temperature and the solar radiation shading temperature. In particular, when the color of the solar radiation shading is dark and the solar radiation absorptance is high, there is a risk that the MRT may increase due to the high temperature object above the human body^12^. Evaporating solar radiation shadings have been proposed as a countermeasure^8^. However, since white solar radiation shades are generally used in most cases, this study does not consider changes in infrared radiation and diffuse solar radiation, including the effects of higher temperatures on solar radiation shades. The relationship between the solar radiation shading rate and SET* and WBGT deductions on representative days is shown in Fig. 8.

### Mist spraying

The effect of air temperature decrease and humidity increase due to mist spraying depends on mist spray amount, which has a constant change in enthalpy, so that the wet bulb temperature Tw in Eq. (4) does not change. According to Eq. (5), MRT does not change but Tg decreases with decreasing of air temperature Td. It is difficult to identify the thermal environment improving effect because it varies according to the distance between the mist spray position and the human body and the wind velocity. Assuming that the human body approaches the mist spray position, air temperature decreases by 2 °C and the corresponding humidity increases by 0.8 g/kg'. Similarly, SET* and WBGT deductions when air temperature decrease and humidity increase are changed correspondingly is shown in Fig. 8.

### Surface cover improvement

Surface temperature on not only the ground but also the wall surface is calculated from Eq. (2). Because it is complicated to assume a building, which requires consideration of the orientation of the wall surface, only the ground surface is assumed. Assuming that the view factors of the sky and the ground are each 0.5, MRT decrease due to the ground surface temperature decrease is calculated using Eq. (3). Surface temperature reduction due to surface cover improvement varies depending on the amount of change in solar reflectance and evaporation efficiency, as well as the weather conditions. For example, Takebayashi et al.^13^ analyzed the relationship between solar reflectance, evaporation efficiency and surface heat budget by experiments on the rooftop and heat budget calculations. SET* and WBGT deductions when surface temperature reduction is changed is shown in Fig. 8.

## Data Availability

The data that support the findings of this study are available from the Osaka Prefectural Institute of Environment, Agriculture, Forestry and Fisheries but restrictions apply to the availability of these data, which were used under license for the current study, and so are not publicly available. Data are however available from the authors upon reasonable request and with permission of the Osaka Prefectural Institute of Environment, Agriculture, Forestry and Fisheries.
